# Network disruption based on multi-modal EEG-MRI in α-synucleinopathies

**DOI:** 10.3389/fneur.2024.1442851

**Published:** 2024-08-22

**Authors:** Chunyi Wang, Jiajia Hu, Puyu Li, Ming Zhang, Liche Zhou, Ningdi Luo, Xue Zhu, Qianyi Yin, Min Zhong, Xinyi Zhou, Hongjiang Wei, Yuanyuan Li, Biao Li, Jun Liu

**Affiliations:** ^1^Department of Neurology and Institute of Neurology, Ruijin Hospital, Shanghai Jiao Tong University School of Medicine, Shanghai, China; ^2^Department of Nuclear Medicine, Ruijin Hospital, Shanghai Jiaotong University School of Medicine, Shanghai, China; ^3^School of Biomedical Engineering, Shanghai Jiao Tong University, Shanghai, China; ^4^Co-innovation Center of Neuroregeneration, Nantong University, Nantong, China

**Keywords:** electroencephalography, magnetic resonance imaging, α-synucleinopathies, isolated rapid eye movement behavior disorder, functional connectivity

## Abstract

**Background:**

Brain network dysfunction has been characterized by resting-state electroencephalography (EEG) and magnetic resonance imaging (MRI) in the prodromal stage. This study aimed to identify multi-modal electrophysiological and neuroimaging biomarkers for differential diagnosis in synucleinopathies and phenoconversion in isolated rapid eye movement sleep behavior disorder (iRBD).

**Methods:**

We enrolled 35 patients with multiple system atrophy (MSA), 32 with Parkinson's disease (PD), 30 with iRBD and 30 matched healthy controls (HC). Power spectral density (PSD) was calculated in different frequency bands. EEG functional connectivity (FC) was calculated using the weighted Phase Lag Index (wPLI) after source localization. Significant network disruptions were further confirmed by MRI FC analysis.

**Results:**

Quantitative EEG analysis demonstrated that delta and theta power spectral density significantly differed among MSA, PD and HC. The increased PSD was correlated with cognitive decline and olfactory dysfunction in PD. Band-specific FC profiles were observed in theta, alpha, and gamma bands. The hypoconnected alpha network significantly correlated with motor dysfunction, while the gamma FC distinguished PD from MSA. By integrating EEG and MRI network analyses, we found that FC between the olfactory cortex and dorsolateral prefrontal cortex was significantly different between MSA and PD. A multimodal discriminative model for MSA and PD, integrating spectral and FC attributes of EEG and MRI, yielded an area under the receiver operating characteristic curve of 0.900. Simultaneously, we found the FC abnormalities were more prominent than spectral features in iRBD indicating prodromal dysfunction. The decreased FC between the angular gyrus and striatum was identified in α-synucleinopathies. This hypoconnectivity was associated with dopaminergic degeneration in iRBD examined by dopamine transporter imaging.

**Discussion:**

Our study demonstrated EEG spectral and functional profiles in prodromal and clinical-defined synucleinopathies. Multimodal EEG and MRI provided a novel approach to discriminate MSA and PD, and monitor neurodegenerative progression in the preclinical phase.

## Introduction

Multiple system atrophy (MSA) and Parkinson's disease (PD) are pathologically and clinically heterogeneous α-synucleinopathies. The pathological hallmark of MSA is the accumulation of α-synuclein within oligodendrocytes, while PD is characterized by pathologic aggregations of α-synuclein in the neurons of substantia nigra, known as Lewy bodies and Lewy neurites (1, 2). The phenotypes of both entities may overlap, making it challenging to differentiate MSA from PD, especially MSA-P subtype (3, 4). Accurate differential diagnosis of PD and atypical Parkinsonism is of paramount therapeutic and prognostic importance. As one of the most specific heralds of synucleinopathy, more than 80% of patients with isolated rapid eye movement (REM) sleep behavior disorder (iRBD) experience phenoconversion over longitudinal follow-up (5). As a prodromal state, iRBD can occur independently with an interval of over 5–15 years before phenoconversion. Patients with iRBD are speculated to exhibit neurodegenerative manifestations concerning clinical symptoms, electroencephalographic slowing, neurodegeneration of sublaterodorsal tegmental nucleus and abnormalities of dopamine transporter (DAT) uptake (6, 7).

Alteration in the functional network may precede structural damage, which can be delineated reliably and reproducibly by functional connectivity (FC) during rest. Aside from magnetic resonance imaging (MRI), increasing interest has been prompted on electroencephalography (EEG) to extract the spectral power and FC characteristics of different frequency bands, providing spectral signatures of cortical oscillations and inter-regional synchronization of brain activity. Diffuse EEG slowing has been identified in PD with mild cognitive impairment and PD dementia (8, 9). Convergent evidence has illustrated the involvement of theta and alpha oscillations not only in cognitive decline but also in motor symptoms and subtypes of PD (10–13). Band-specific characteristics of electroencephalographic FC have been reported in PD with a gradual dysconnectivity in the alpha band (14), which is associated with motor dysfunction. A recent study demonstrated that PD patients presented significantly higher delta and theta power and slow-to-fast ratio in the temporal and occipital regions than MSA, and clarified the enhanced FC of delta and theta bands in the posterior region in MSA instead of frontal regions in PD (15). However, the discriminative power is limited. Studies have also recognized the cortical disruptions in iRBD by EEG slowing, slow wave activity, sleep spindles and K-complexes during sleep and wakefulness, which are associated with the level of cognitive impairment and profitable for the identification of phenoconverison (13–17). A resting-state EEG study found decreased delta-band FC in the frontal brain regions of iRBD (18). Nevertheless, the disruptions of electroencephalographic network in MSA and iRBD are not well understood currently due to the limitation of scalp EEG. Given the advances in cortical source reconstruction method, we implemented source localization using the method of standardized low-resolution electromagnetic tomography (sLORETA) to trace intracranial electric neuronal activity, instead of calculating the head-surface EEG signals of electric potential difference. The weighted phase lag index (wPLI) was computed based on phase synchronization to alleviate volume conduction and noise disturbance. This approach provided compensatory measures of intrinsic connectomics aside from MRI and oscillatory activity in certain frequency bands. In PD patients, the network-specific changes of functional connectome in sensorimotor network (SMN), default mode network (DMN) and basal ganglia (BG) are demonstrated to correlate with motor severity (16, 17). Compared to PD, MSA patients showed reduced cerebellar FC with the striatum and other networks including the frontoparietal network (FPN), SMN, salience network and DMN (18, 19). The severity of MSA is correlated negatively with cerebellar FC. Seed-based network analysis mainly focuses on the BG circuitry to detect early dysfunction in patients with iRBD (20). Whole brain analysis revealed disrupted connectivity in associative regions of the temporal and parietal lobes that supported complex cognitive functions in iRBD (21). Thus, it is important to reveal the heterogeneous network disruptions in different phases and directions of synucleinopathy.

Since a single modality may lead to biased estimates with a small sample size, current results are conflicting to recapitulate which may be attributed to inconsistent methodologies. We integrated EEG and MRI functional networks to substantiate the intrinsic intercommunication patterns of neural networks implying the symptoms, disease processes, and differential diagnosis. Multimodal fusion approaches can significantly improve the spatiotemporal resolution limited by any single modality. In general, our study aimed to: (1) investigate electroencephalographic characteristics in α-synucleinopathies in aspect of spectral power and FC, as well as their band-specific correlation with clinical symptoms; (2) reveal divergent electroencephalographic profiles of MSA and PD and construct EEG-MRI multimodal model for the differential diagnosis; (3) reveal specific aberrant EEG network in iRBD to provide biomarkers for early prediction.

## Materials and methods

### Participants

A total of 35 patients with MSA, 32 with PD, 30 with iRBD and 30 healthy individuals matched by age, sex, and educational level were enrolled between Jan 2022 and July 2023 from Ruijin Hospital. All subjects were right-handed. Clinically established MSA or clinically probable MSA was diagnosed according to the International Parkinson and Movement Disorder Society (MDS) Criteria for the Diagnosis of MSA (22). Patients with idiopathic PD were diagnosed according to the MDS diagnostic criteria (23). The diagnosis of iRBD was made based on the third edition of the International Classification of Sleep Disorders criteria. The presence of REM sleep without atonia and dream enactment behavior was acknowledged by video-polysomnography in all patients with iRBD. Individual informed consent was obtained in accordance with the Declaration of Helsinki, and the study was approved by a local ethics committee. Clinical features and demographics of all subjects are summarized in Table 1.

**Table 1:** Demographic and clinical characteristics of the participants.

**Characteristics**	**MSA**	**PD**	**iRBD**	**HC**	***P* value**
**Demographics**					
Gender, male%	60.0 (21/35)	56.3 (18/32)	63.3 (19/30)	46.7 (14/30)	0.309
Age, year	60.9 (5.6)	65.8 (8.2)	65.8 (5.7)	63.3 (8.7)	0.067
Education, year	10.1 (3.5)	12.1 (4.0)	12.2 (2.8)	11.2 (3.6)	0.187
**Clinical features**					
Disease duration (month)	33.4 (20.8)	60.8 (38.5)	57.5 (37.9)	-	**0.002**
MoCA	20.5 (6.9)	23.6 (4.7)	24.5 (2.5)	26.3 (2.6)	**< 0.001**
MMSE	24.2 (6.4)	27.3 (3.1)	28.1 (1.7)	28.5 (1.5)	**< 0.001**
NMSS	10.7 (4.2)	7.5 (4.4)	5.1 (2.1)	1.7 (1.9)	**< 0.001**
SCOPA-AUT	21.2 (10.1)	10.3 (8.2)	5.5 (3.4)	2.7 (2.5)	**< 0.001**
HAMD17	10.3 (5.9)	7.1 (5.5)	3.2 (2.1)	0.9 (1.1)	**< 0.001**
HAMA	12.6 (6.5)	8.8 (6.2)	3.7 (2.3)	1.0 (1.2)	**< 0.001**
RBDSQ	4.4 (3.2)	3.6 (3.1)	7.8 (1.8)	0.4 (1.0)	**< 0.001**
SS-16	8.3 (2.9)	6.5 (3.1)	7.0 (3.9)	11.2 (3.8)	**< 0.001**
UPDRS.I	12.8 (6.7)	8.3 (4.9)	4.6 (2.1)	-	**< 0.001**
UPDRS.II	20.5 (11.7)	11.9 (5.5)	0.4 (0.8)	-	**< 0.001**
UPDRS.III	38.7 (21.1)	31.4 (14.2)	0.1 (0.2)	-	**< 0.001**
UMSARS.I	16.4 (8.7)	-			
UMSARS.II	19.5 (9.4)	-			
UMSARS.IV	2.9 (1.5)	-			

MoCA, Montreal Cognitive Assessment; MMSE, Mini-Mental State Examination; SCOPA-AUT, Scales for Outcomes in Parkinson's Disease-Autonomic Dysfunction; NMSQ, Non-Motor Symptoms Questionnaire; HAMA, Hamilton Anxiety Scale; HAMD, Hamilton Depression Scale; RBDSQ, RBD screening questionnaire; SS-16, Sniffin' Sticks Identification Test 16; UPDRS, Unified Parkinson's Disease Rating Scale; UMSARS, Unified Multiple System Atrophy Rating Scale. Bold values denote statistical significance among groups.

### EEG recording and preprocessing

Scalp EEG of all the participants was recorded using a 64-channel EEG recording system by Be Plus PRO amplifier (EB NEURO, Florence, Italy) with electrodes positioned according to the international 10–20 system. The impedances of all electrodes were kept below 5 kΩ. The monitoring of the eye movements was obtained from vertical and horizontal EOGs. Resting-state EEG was recorded for a total of 10 minutes while all participants were relaxed and awake with their eyes closed in a sound-attenuated room. Data were analyzed with MATLAB R2011b software using scripts from the EEGLAB 2020.0 toolbox (24). Details regarding EEG processing are provided in Supplementary material.

### EEG source reconstruction

Preprocessed recordings were later imported into Brainstorm toolbox for source reconstruction (http://neuroimage.usc.edu/brainstorm). The Boundary Element Method (BEM) as implemented in OpenMEEG was used to compute the forward head model using Brainstorms default parameters with a Montreal Neurological Institute (MNI) MRI template (ICBM152). We then apply the method of sLORETA to obtain plausible EEG source estimates. We proceeded to divide the brain into 90 cortical and subcortical regions of interest (ROIs) according to the Anatomical Automatic Labeling (AAL) atlas (25).

### Power spectral density (PSD), FC analysis and graph theory

PSD is the most common EEG feature to provide the power distribution of the EEG series in the frequency domain (26). We performed the spectral analysis of the EEG data in EEGLAB functions. The PSD was estimated using Welch's method by Fast Fourier Transform (FFT) algorithm, with a 2-second Hanning window and a 50% overlap. Absolute PSD was calculated in the following frequency bands: delta (1–4 Hz), theta (4–8 Hz), alpha (8–13 Hz), beta (13–30 Hz), gamma (30–60 Hz).

Functional connectivity denotes statistical dependency between time series of physiological signals recorded from different brain regions (27). wPLI was calculated to measure phase synchronization weighted by the magnitude of the imaginary component of the cross-spectrum. The instantaneous phase for each time point was calculated using the Hilbert transform. We generated global connectivity strength by averaging the wPLI over all electrode pairs in different frequency bands. A groupwise comparison of global EEG FC was implemented between two groups of patients. Regions with higher degree were defined as ROIs to investigate the connections with whole brain regions. Results were visualized using the BrainNet Viewer.

The brain network is consisted of nodes (vertices) and links (edges) between pairs of nodes. Nodes usually denote brain regions, while links represent functional connections (28). Graph topology of whole-brain resting-state EEG can be quantitatively analyzed by a wide variety of graph theoretic measures, which provide a methodology to depict complex interactions between large-scale networks. Three conspicuous graph theory attributes for reconstructed graphs including global efficiency (Eglob), local efficiency (Eloc), modularity and nodal degree were estimated. Eglob represents average inverse shortest path length between all pairs of nodes in the network. Eloc denotes Eglob computed on the node's neighbors (29). Complex network is composed of a number of modules. Higher network modularity tends to form more dense connections within modules and sparser connections between modules (30). Nodal degree represents the number of connections that link to a node (28). Group comparisons of the wPLI were performed to localize different functional connections between groups.

### MRI and PET acquisition

MRI of all the participants was acquired with 3T Siemens scanners with a 12-channel head coil. The scanning protocol included a high-resolution three-dimensional structural T1-weighted magnetization prepared rapid acquisition gradient-echo (MPRAGE) sequence acquired sagittally and a whole-brain echo-planar imaging (EPI) run sensitive to blood oxygen level dependent (BOLD) contrast.

DAT imaging substantiates the severity of neurodegeneration in iRBD in the prodromal stage. ^18^F-FP-CIT exhibits a high affinity for the DAT with high diagnostic accuracy and clinical convenience. We intended to investigate whether the mechanism of brain electrophysiological disruption is associated with dopaminergic deficit in order to identify potential biomarkers. DAT imaging of a subset of 14 iRBD patients was collected. ^18^F-N-(3-fluoropropyl)-2b-carbon ethoxy-3b-(4-iodophenyl) nortropane (^18^F-FP-CIT) hybrid PET/MRI examinations were performed on Siemens Biograph mMR scanner (Siemens Healthcare) using an 8-channel phase-array head coil to estimate DAT deficit in patients with iRBD. During data collection, subjects were instructed to keep their eyes closed, relax but awake. Details regarding the MRI and PET acquisition and preprocessing are provided in Supplementary material.

### MRI FC analysis

To verify the discrepancies detected from group comparisons and matrices of EEG connectivity, we conducted a seed-to-seed based inter-regional correlation analysis. Based on the groupwise comparisons of EEG FC, significant FC alterations were identified between groups. We selected a collection of regions presenting the most significant difference as a cluster mask and defined it as ROI for MRI FC analysis. The following masks were created according to the AAL atlas in standard space: left and right dorsolateral prefrontal cortex (DLPFC; AAL 7+11+13 and 8+12+14), basal ganglia including left and right striatum (AAL 71+73 and 72+74) and left and right amygdala (AAL 41 and 42).

### PET ROI analysis

**Figure 1:**
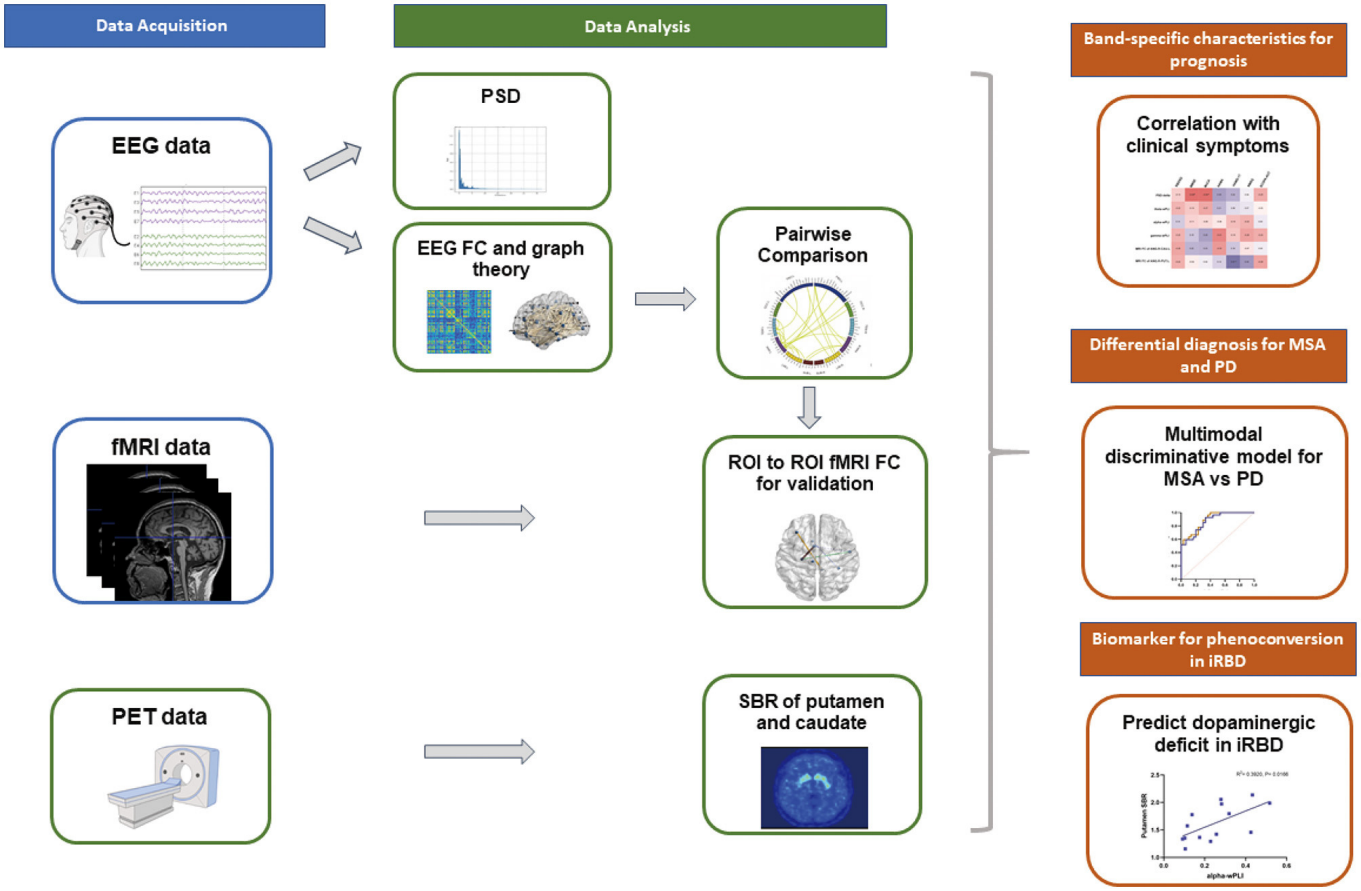
Schematic of the multimodal analysis pipeline. High-density EEG recordings were collected in patients with iRBD, MSA, PD and HC. Quantitative EEG analysis including spectral analysis, FC and graph theory were computed after source localization. Group comparisons suggested specific network disruptions which were verified by subsequent fMRI analysis. Band-specific EEG profiles significantly correlated with clinical manifestations. Spectral power, EEG and fMRI connectivity were integrated to establish a reliable discriminative model for synucleinopathies. EEG and MRI networks also identified biomarkers of functional abnormalities in iRBD, MSA and PD. ^18^F-FP-CIT PET data were collected from patients with iRBD to reveal the relationship between DAT deficit and EEG indices.

^18^F-FP-CIT PET was implemented to evaluate DAT availability. Caudate and putamen were set as target regions of interest. The left and right occipital cortices were administrated as reference regions. Striatal binding ratios (SBRs) were calculated as [(target region/reference region) - 1]. The average of right and left putamen, left and right caudate were taken as the total caudate SBR and total putamen SBR respectively. The MRI template incorporating these ROIs was loaded onto the PET scan of each patient. Figure 1 showed the radiomics workflow.

### Statistical analysis

Analysis was performed with the SPSS 20.0 (SPSS Inc., Chicago, IL, USA). Group comparisons of numerical variables were performed using either analysis of variance (ANOVA) for normal data or Kruskal-Wallis test for nonnormal data. Sex distribution was compared using a χ2 test. Correlations between EEG and DAT SBR/clinical scales were performed using Spearman's correlation. *P*-values were adjusted for multiple comparisons using the false discovery rate (FDR) method. A two-tailed *p* < 0.05 after the multiple testing corrections was considered statistically significant. We implemented binary logistic regression and receiver operating characteristic (ROC) analysis to test for the potential utility of the most significant markers, setting gender, age, levodopa equivalent daily dose, and disease duration as covariates. Corresponding AUC was calculated to quantify their discriminative power.

## Result

### Demographic and clinical characteristics

The disease duration of MSA patients was shorter compared to PD and iRBD groups. Significant RBD symptoms were presented in iRBD instead of MSA and PD. Severe motor disorders were present bilaterally in MSA and PD. Detailed clinical scores and corresponding statistical analysis among MSA, PD, iRBD and HC groups are listed in Table 1.

### PSD analysis in patients with MSA and PD

**Figure 2:**
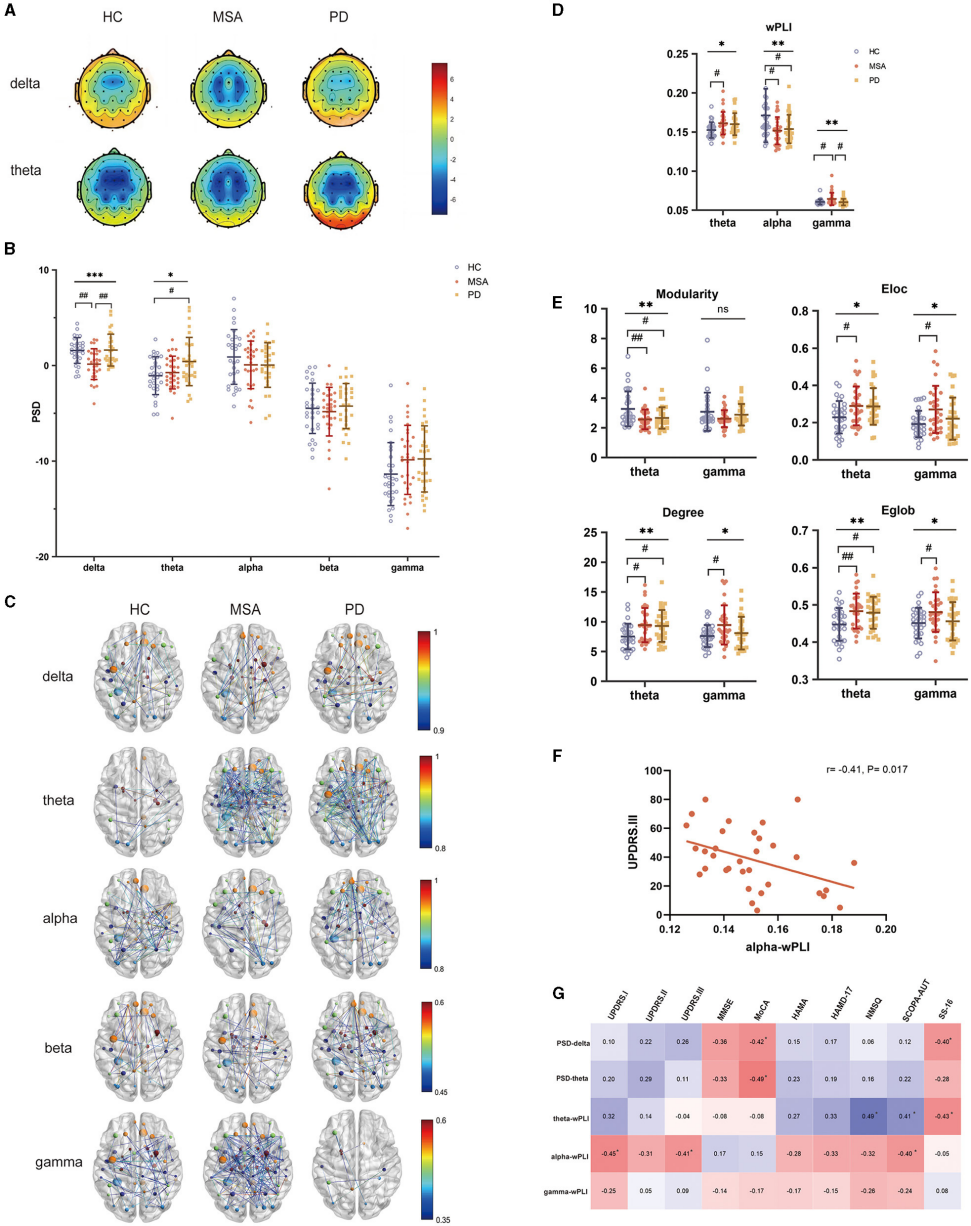
PSD and FC attributes of EEG analysis in HC, MSA and PD. **(A)** Topological maps representing the global PSD per group within the delta and theta band. **(B)** Comparison of global PSD in different frequency bands among HC, patients with MSA and PD. **(C)** EEG FC analysis after source reconstruction across different frequency bands and groups. **(D)** wPLI in the theta, alpha and gamma bands showed significant differences. **(E)** Graph theory of modularity, Eloc, degree, Eglob in the theta and gamma bands showed significant differences. **(F)** Relationship between alpha wPLI and UPDRS.III in MSA. **(G)** Relationship between EEG indices and the severity of motor and nonmotor dysfunctions in PD. One-way ANOVA: ^*^p < 0.05, ^**^p < 0.01, ^***^p < 0.001. *Post-hoc* tests corrected for multiple comparisons: ^#^*p* < 0.05, ^##^*p* < 0.01.

The PSD across all scalp electrodes was calculated in delta, theta, alpha, beta and gamma bands to quantify EEG power spectra (Figures 2A, B). In comparison with HC, the delta power was remarkably decreased in patients with MSA, while increased in PD (F = 8.8, *P* = 0.00033). After multiple comparison analysis, significantly higher delta PSD was demonstrated in patients with PD when comparing with MSA (*P* = 0.0015). MSA exhibited lower delta PSD comparing with HC (*P* = 0.0014). Comparing with HC, patients with PD showed increased PSD in the theta band, while the increase in MSA is not significant (F= 4.1, *P* = 0.019; PD vs. HC: *P* = 0.020).

### Whole brain EEG-FC analysis and network properties in patients with MSA and PD

To assess the interaction of the brain functional network derived from electrophysiological examination, wPLI was calculated to represent the connectivity strength (Figure 2C). The FC of MSA, PD and HC differed in the theta, alpha and gamma bands (Figure 2D). Compared with HC, wPLI in the theta band was significantly increased in patients with MSA (F = 4.0, P = 0.022; MSA vs. HC: *P* = 0.029). Besides, we observed decreased alpha wPLI both in MSA and PD compared with HC (F = 4.9, *P* = 0.0097; MSA vs. HC: *P* = 0.019; PD vs. HC: *P* = 0.026). Gamma wPLI significantly increased in MSA compared with HC and the robustness of FC in the gamma band for distinguishing MSA from PD was underlined after multiple comparison correction (F = 5.0, *P* = 0.0091; MSA vs. HC: *P* = 0.029; MSA vs. PD: *P* = 0.011). No significant discrepancies were detected from other frequencies of bands.

The graph theory probed the topology of EEG interrelatedness at the source pair (Figure 2E). The evaluation of modularity within the theta range showed a significant decrease in PD and MSA (F = 5.6, *P* = 0.0051). The increase of theta-band Eloc, degree and Eglob was also detected in MSA and PD (Eloc: F = 3.8, *P* = 0.026; degree: F = 4.9, *P* = 0.0092; Eglob: F = 5.7, *P* = 0.0046). In terms of Eloc, degree and Eglob in the gamma band, there was an accordant increase in MSA and PD (Eloc: F = 4.2, *P* = 0.019; degree: F = 4.0, *P* = 0.022; Eglob: F = 3.1, *P* = 0.048).

### Band-specific EEG indices associated with clinical manifestations

Within-group Spearman's correlation analysis revealed band-specific associations with clinical manifestations. For patients with MSA, alpha-band wPLI were negatively correlated with UPDRS-III (r = −0.41, *P* = 0.017) (Figure 2F). For patients with PD, delta- (MMSE: r = −0.36, *P* = 0.048; MoCA: r = −0.42, *P* = 0.028) and theta- band PSD (MoCA: r = −0.49, *P* = 0.009) showed a negative correlation with cognitive performance. Increased PSD in the delta band also correlated with olfactory dysfunction (r = −0.40, *P* = 0.037). Theta band wPLI also exhibited a relationship with NMSQ (r = 0.49, *P* = 0.009) and olfactory function (r = −0.43, *P* = 0.024). Alpha-band wPLI negatively associated with UPDRS.I (r = −0.45, *P* = 0.020) and UPDRS.III (r = −0.41, *P* = 0.035) (Figure 2G).

### FC between olfactory cortex (OLF) and DLPFC significantly differed between MSA and PD

**Figure 3:**
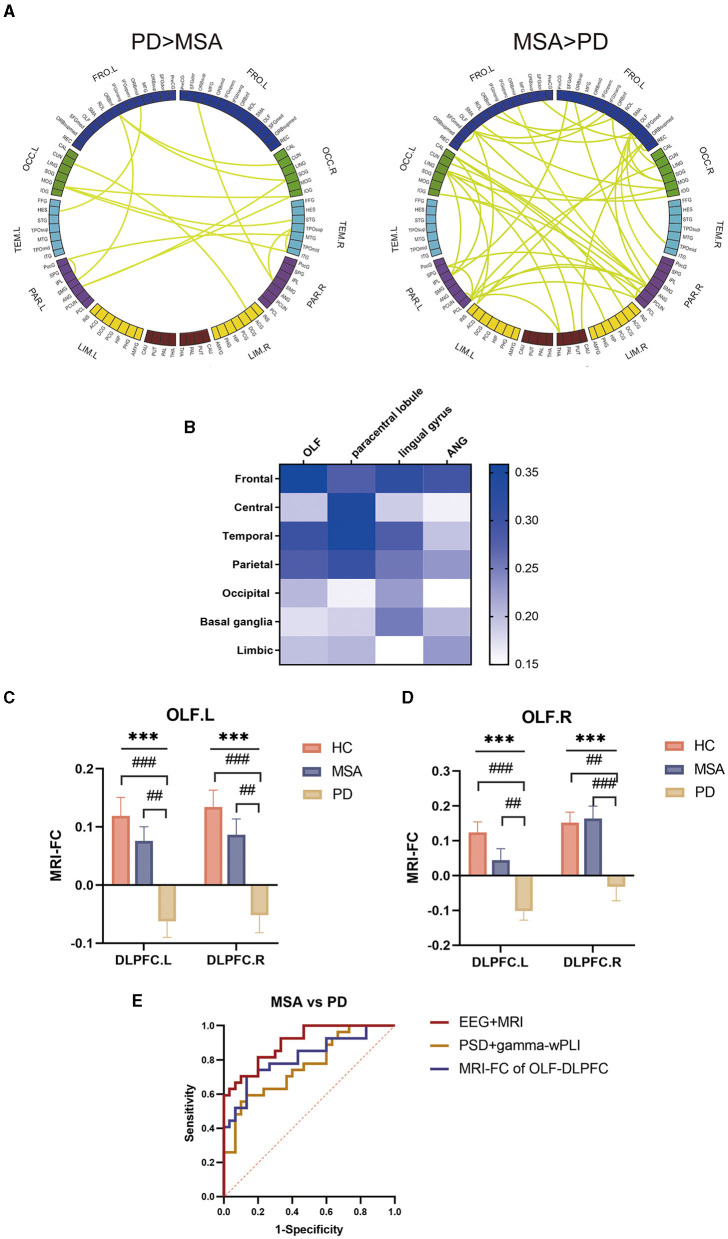
Groupwise comparisons of whole brain EEG FC and corresponding MRI analysis between MSA and PD. **(A)** Circular graph visualizing the brain regions responsible for discrepant EEG FC between MSA and PD. **(B)** Matrices presenting altered FC between ROIs and whole brain regions in the gamma band. **(C)** MRI FC analysis between OLF.L and DLPFC. **(D)** MRI FC analysis between OLF.R and DLPFC. **(E)** ROC curves presenting the multimodal discriminative model for MSA vs. PD with high sensitivity and specificity.

Since preliminary global EEG-FC of MSA and PD significantly differed in the gamma band after *post hoc* analysis. We went ahead to implement a groupwise comparison of global EEG FC between MSA and PD (Figure 3A). Regions with higher degrees were located in bilateral OLF, left paracentral lobule, left lingual gyrus and right ANG. The regions above were defined as ROIs bilaterally to investigate the connections with whole brain regions. The major difference in FC was located in OLF which exhibited prominent connections with frontal lobes (Figure 3B).

The significant difference of FC between OLF and DLPFC was confirmed by seed-to-seed MRI analysis in MSA and PD. The FC between OLF.L and DLPFC.L (F = 11.4, *P* = 0.00041; MSA vs. PD: P = 0.002030; PD vs. HC: *P* = 0.000054) and between OLF.L and DLPFC.R (F = 11.2, *P* = 0.00047; MSA vs. PD: *P* = 0.00266; PD vs. HC: *P* = 0.000055) significantly differed (Figure 3C). As for OLF.R, it showed significantly different connections with DLPFC.L (F = 12.7, *P* = 0.00003; MSA vs. PD: *P* = 0.00212; PD vs. HC: *P* = 0.00002) and DLPFC.R (F = 9.3, *P* = 0.00024; MSA vs. PD: *P* = 0.000610; PD vs. HC: *P* = 0.00166) among the three groups (Figure 3D).

Based on the above analysis, EEG metrics and MRI FC were integrated to improve the discriminative power in patients with PD and MSA (Figure 3E). A combination of delta PSD, gamma wPLI as well as MRI FC between bilateral OLF and DLPFC yielded a satisfying predictive value for MSA vs. PD with an area under the ROC curve (AUC) of 0.900 (95%CI 0.824- 0.976; sensitivity: 0.815; specificity: 0.800).

### Hypoconnected networks of parietal subregions in patients with MSA and PD

**Figure 4:**
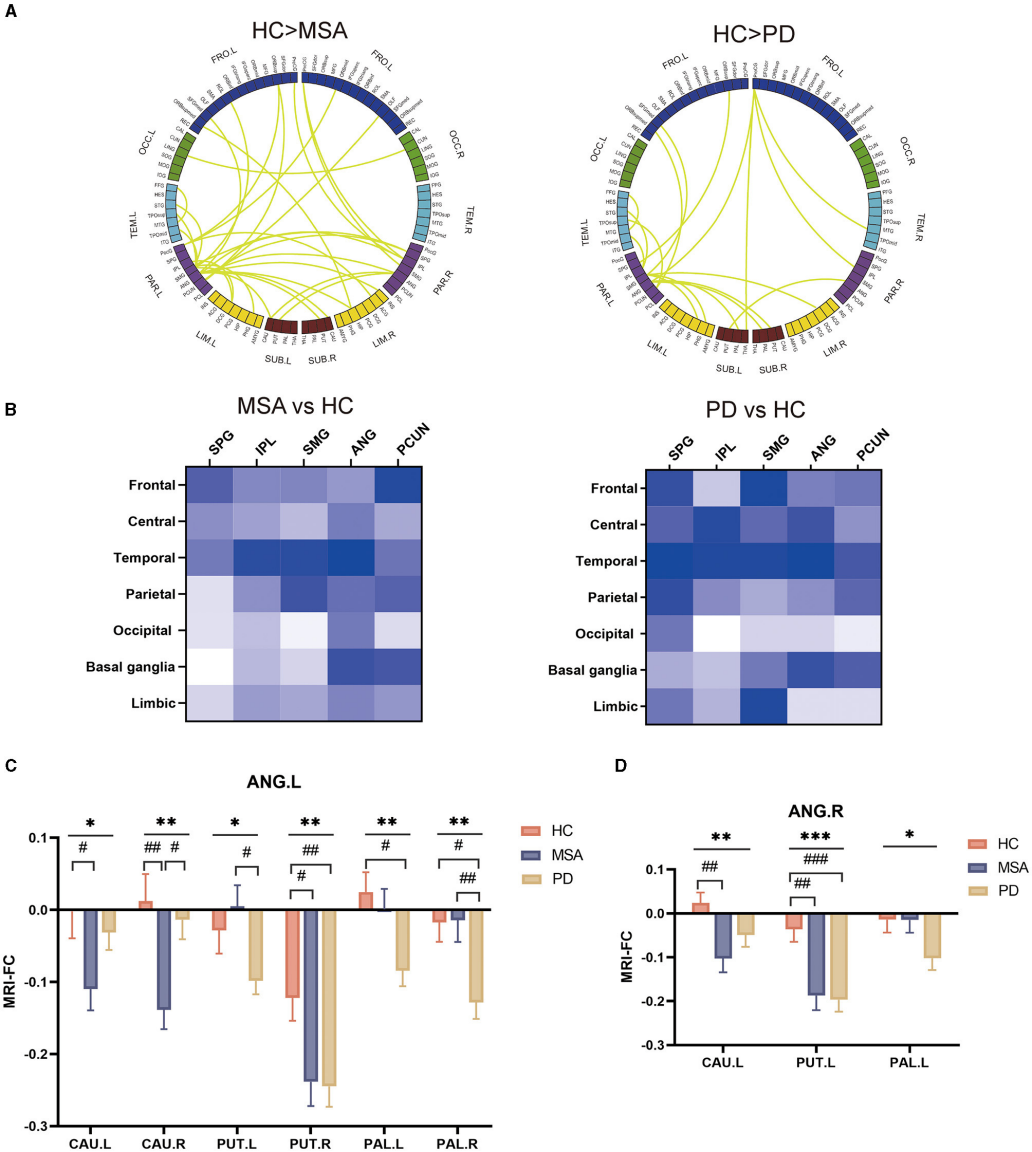
Groupwise comparisons of whole brain EEG FC and corresponding MRI analysis in patients with MSA and PD, and their clinical correlates. **(A)** Circular graph visualizing the brain regions responsible for the between-group EEG-FC discrepancies. **(B)** Matrices presenting altered FC between parietal lobe subregions and whole brain regions in the alpha band. **(C)** MRI FC analysis between ANG.L and basal ganglia. **(D)** MRI FC analysis between ANG.R and basal ganglia.

Interestingly, according to *post hoc* analysis, we found that the alpha band EEG FC significantly descended in both MSA and PD patients. Furthermore, we implemented group comparisons of the connectivity matrix derived from EEG analysis in the alpha band to localize the neural activity responsible for the discrepancies between synucleinopathies and healthy individuals, respectively. As shown in Figure 4A, the connectivity involving the parietal lobe was remarkably reduced in MSA and PD. To further identify the parietal subregions that were most implicated in reduced FC, parietal subregions with higher degrees including superior parietal gyrus (SPG), inferior parietal but supramarginal and angular gyri (IPL), supramarginal gyrus (SMG), angular gyrus (ANG), precuneus (PCUN) was defined as the ROI bilaterally (Figure 4B). The major of reduced FC with basal ganglia was located in ANG.

To confirm the functional abnormalities, those parietal subregions served as bilateral seeds for subsequent ROI-to-ROI MRI-FC analyses with bilateral caudate (CAU), putamen (PUT), pallidum (PAL) and amygdala (AMYG). We found that ANG.L showed significantly altered FC with CAU (CAU.R: F = 6.2, *P* = 0.003; CAU.R: F =3.8, *P* = 0.026), PUT (PUT.L: F = 3.8, *P* = 0.026; PUT.R: F =4.7, *P* = 0.011), PAL (PAL.L: F = 3.9, *P* = 0.023; PAL.R: F = 4.9, *P* = 0.0098). Compared with HC, the altered FC was more prominent between ANG and CAU, and between ANG and PUT in patients with MSA (FC of ANG.L-CAU.L: *P* = 0.040; FC of ANG.L-CAU.R: *P* = 0.007; FC of ANG.L-PUT.R: *P* = 0.029; FC of ANG.R- CAU.L: *P* = 0.006; FC of ANG.R- PUT.L: *P* = 0.002) (Figure 4C). In relative to HC, patients with PD showed remarkably reduced FC between ANG and PUT, and between ANG and PAL (FC of ANG.L-PUT.R: *P* = 0.020; FC of ANG.L-PAL.L: *P* = 0.036; FC of ANG.L-PAL.R: *P* = 0.027; FC of ANG.R- PUT.L: *P* = 0.00082) (Figure 4D).

### Whole brain EEG profiles and hypoconnected parietal-striatal brain network in iRBD

Since both MSA and PD patients showed specific spectral profiles and hypoconnectivity, we speculated that it might be also characterized in iRBD to identify promising biomarkers for phenoconversion. We found the PSD did not show a significant difference compared with HC in any frequency of bands (Supplementary Figures 1A, B). Notably, slight FC alteration was observed in the theta band in iRBD (t = 2.3, *P* = 0.026) (Supplementary Figures 1C, D). Corresponding graph theory attributes including degree, Eloc and Eglob showed slightly abnormalities in the theta band as well (degree: t = 2.0, *P* = 0.046; Eloc: t = 2.3, *P* = 0.026; Eglob: t = 2.0, *P* = 0.046) (Supplementary Figure 1E). Correlation analysis revealed significant relationship between delta and cognitive performance in iRBD (MMSE: r = −0.54, *P* = 0.018; MoCA: r = −0.4, *P* = 0.018). Besides, we also observed the relationship between delta PSD and olfactory function in iRBD (r = −0.55, *P* = 0.015) (Supplementary Figure 1F).

**Figure 5:**
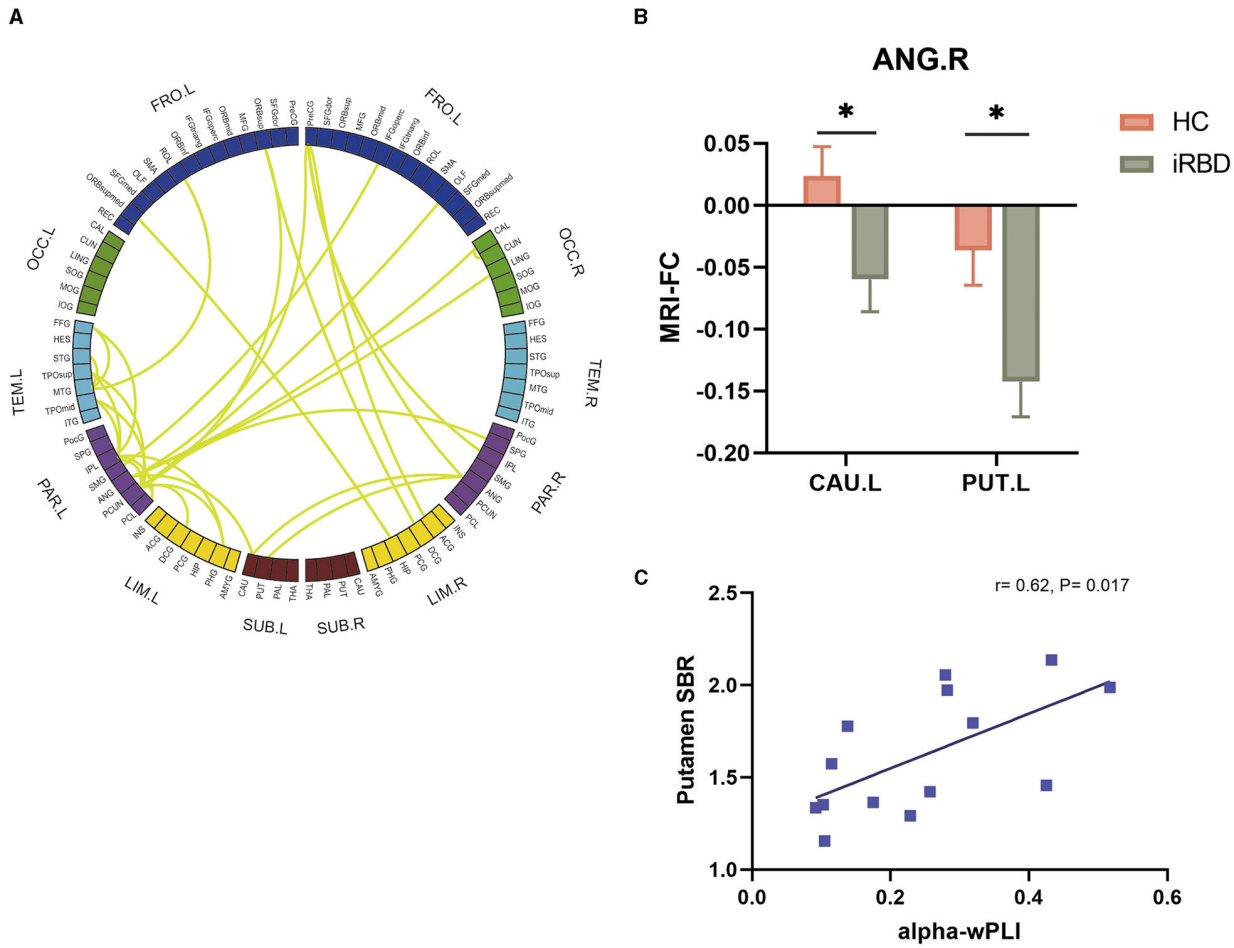
Groupwise comparisons of whole brain EEG FC and corresponding MRI analysis in patients with iRBD, and the correlation between alpha wPLI and DAT deficit. **(A)** Circular graph visualizing the brain regions responsible for the EEG-FC discrepancies comparing iRBD with HC. **(B)** MRI FC analysis between ANG.R and striatum. **(C)** Correlation between putamen SBR and EEG FC between ANG and striatum in the alpha band.

The decline of global EEG FC in the alpha band and association with motor dysfunction were observed in patients with MSA and PD. Simultaneously, our analysis also revealed homogeneous alterations of brain network in iRBD (Figure 5A). It was characterized by the hypoconnectivity of the parietal lobe. MRI FC analysis further confirmed the aberrant parietal-striatal connectivity. We detected decreased FC between ANG.R and CAU.L (t = −2.4, *P* = 0.021), as well as between ANG.R and PUT.L (t = −2.6, *P* = 0.011) (Figure 5B).

To explore the association between EEG-FC and the dopaminergic function in iRBD, 14 patients of the iRBD group underwent DAT-PET/MRI scan. Spearman correlation analysis revealed that there were significant negative correlations between DAT SBR in the putamen and EEG-FC between ANG and striatum (r = 0.62, *P* = 0.017) (Figure 5C).

### FC alterations between iRBD and clinically diagnosed α-synucleinopathies

To investigate the network disruptions in different directions of phenoconversion, we implemented group comparisons of the EEG connectivity matrix between MSA and iRBD as well as between PD and iRBD. According to the results of global wPLI in different frequency bands, the most significant difference between MSA and iRBD appeared at gamma oscillation (F = 3.8, *P* = 0.026; MSA vs. iRBD: *P* = 0.022) (Supplementary Figure 2A), while the most significant difference between PD and iRBD was at theta oscillation (F = 3.2, *P* = 0.046; PD vs. iRBD: *P* = 0.036) (Supplementary Figure 2B). A groupwise comparison of global EEG FC was implemented between MSA and iRBD in the gamma band (Supplementary Figure 2C) as well as between PD and iRBD in the theta band (Supplementary Figure 2D) showing significant FC alterations. Furthermore, we testified the functional abnormalities in the circular graphs using ROI-to-ROI MRI-FC analyses. Consistent with EEG FC alterations, MRI FC between right superior occipital gyrus (SOG.R) and right fusiform gyrus (FFG.R) was decreased in MSA and iRBD (F= 5.715, *P* = 0.005; HC vs. MSA: *P* = 0.004; MSA vs. iRBD: *P* = 0.048) (Supplementary Figure 2E). Besides, MRI FC between OLF.R and SOG.R was decreased in PD and iRBD (F = 5.715, *P* = 0.007; HC vs. PD: *P* = 0.010; PD vs. iRBD: *P* = 0.033) (Supplementary Figure 2F).

## Discussion

Our study identified the spectral and functional abnormalities revealed by EEG and MRI in patients with α-synucleinopathies. Quantitative EEG analysis revealed the band-specific characteristics in MSA and PD. PSD analysis demonstrated the most significant abnormalities in the delta band which was correlated with cognitive performance in PD and iRBD. Whole brain FC analysis differed in the theta and gamma bands, which exhibited correlations with nonmotor symptoms. And we found that the gamma-band FC was significantly distinct in PD and MSA. The altered FC in the alpha band was remarkably associated with motor symptoms. Furthermore, network-based EEG and MRI analysis identified that connectivity between OLF and DLPFC specifically differed in PD and MSA. Multimodal approaches based on delta PSD, gamma wPLI and MRI FC between OLF and DLPFC presented discriminative potential for MSA vs. PD. In addition, EEG and MRI network analysis revealed aberrant connectivity between ANG and striatum in iRBD, MSA and PD compared with HC, suggesting a homogeneous pathophysiological process. It is promising as a prodromal biomarker, considering the relationship with DAT alterations in iRBD. The combination of EEG and MRI meets the requirement of high temporal and spatial resolution for the complementary study of the brain's electrophysiology and hemodynamics to understand the complexities of cortical function. In that sense, we provided novel methodologies combining EEG and MRI networks to investigate functional abnormalities in α-synucleinopathies.

The spectral power and FC attributes of high-density EEG were evaluated in our study. Spectral features have been extensively acknowledged as promising markers for many neurodegenerative diseases that can lead to dementia (31). EEG slowing is frequent in α-synucleinopathies, especially PD. The EEG measures can also provide an approximation of phosphorylated α-synuclein level in PD cortex (32). A preliminary study found that Lewy type synucleinopathy severity was significantly associated with increased delta-band power (33). In accordance with previous studies, we found that delta PSD of MSA significantly descended while PD exhibited a negligible increase (15). Cholinergic impairment participates in the pathophysiological mechanism of brain electricity slowing in neurodegenerative diseases (34). Our finding indicated more serious synaptic dysfunction and cholinergic deficits in patients with PD. Cognitive decline and olfactory dysfunction are common in PD. Delta PSD seemed to be related with cognitive performance and olfactory function in PD (35).

Furthermore, whole brain FC was implemented for MSA and PD in order to identify band-specific characteristics relevant to clinical manifestations. Studies have demonstrated reduced connectivity in the alpha and beta bands in the early stage of PD preceding the alteration of spectral analysis (36). We detected the altered alpha-band FC prior to the abnormalities of spectral analysis in α-synucleinopathies. Previous studies have discussed that elevated theta and reduced alpha connectivity influenced cognition, visuospatial performance and executive control in patients with PD (12, 14, 16, 37). Besides, pathologically reduced gamma FC was observed in PD, whereas MSA showed the opposite (38). Theta and gamma oscillations are considered to be associated with movement executions (12, 36). Based on the graph theory results, we observed the increase of Eloc, Eglob and degree in theta and gamma band indicating a compensatory mechanism mediated by cortical neurons in the early stage of α-synucleinopathies. It is recognized that those observable abnormalities mostly represented the impact of nonmotor symptoms on the brain network. We observed that theta-band wPLI was significantly associated with the nonmotor symptoms in PD. It is speculated that the theta connectivity is elevated to accommodate for more severe motor and cognitive deficits. In addition, there was a remarkable relationship between alpha-band wPLI and UPDRS.III both in patients with MSA and PD. Alpha reactivity may be mediated by cortically projecting cholinergic nuclei (12, 38). The relationship between alpha FC and motor dysfunction indicated impaired cholinergic transmission (39). Our results suggested that alpha-band FC disruption might reflect motor dysfunction.

The most significant different FC between MSA and PD was observed in the gamma band. We performed a group comparison of wPLI between MSA and PD to reveal the network-based discrepancy in the gamma band. We found that OLF is significantly different showing higher degrees when comparing MSA and PD in the brain network. As is known, olfactory system dysfunction is commonly observed years before the commencement of parkinsonism in PD and dementia with Lewy bodies, whereas it is not prominent in MSA. Olfactory network disruption typically arises in conjunction with cognitive impairment. Focusing on OLF, we found aberrant connections with the prefrontal cortex (PFC). PFC is involved in diverse functions ranging from cognition, emotion and executive action. Subsequent MRI analysis identified the connections between DLPFC and OLF, were significantly different in MSA and PD. In patients with PD, hypoactivated left DLPFC is mainly associated with affective symptoms in neuroimaging studies, while right DLPFC primarily plays a crucial role in executive function. Our results speculated the functional abnormalities between OLF and DLPFC might interpret the heterogenous pathological influence in PD and MSA, and discriminate PD from atypical parkinsonism in the early stage. It is worth further investigation whether the olfactory pathway could affect cognition and execution via modulating PFC circuitry (39). Repetitive transcranial magnetic stimulation (rTMS) targeting left DLPFC has shown alleviation for depression and cognitive impairment in PD (40). rTMS targeting right DLPFC showed therapeutic improvement for the nonmotor and motor symptoms in a recent study (41). We also exploited collaborative EEG and MRI analysis in the differential diagnosis of MSA and PD. The optimal model comprising delta spectral power and gamma EEG FC as well as MRI FC presented high sensitivity and specificity.

iRBD is extensively acknowledged as a powerful sign of imminent neurodegeneration. Preliminary EEG analysis demonstrated the slowing of the power spectrum and decreased FC toward lower frequencies as a preclinical index (42, 43). It is speculated as an active compensatory mechanism of cognitive impairment in the prodromal stage of synucleinopathies (44). Nevertheless, we did not observe significantly aberrant PSD regardless of the relationship between delta PSD and cognitive performance as well as olfactory function in iRBD. Instead of PSD, we speculated that the disrupted FC might characterize the disease progression in the early stage. Network-based FC changes are associated with progressive pathophysiology, which starts years before the onset of motor dysfunctions. Alpha-band FC was significantly correlated with motor dysfunction. Groupwise EEG FC contrasts localized the significantly divergent network connectivity in parietal areas in the alpha band for MSA, PD and iRBD relative to HC. Motor function is under the manipulation of the extensive regions involving frontal, parietal, and temporo-occipital areas. Previous studies clarified the metabolic decrease in the parietal cortex in PD as well as MSA patients (45). In patients with iRBD, it is reported that parietal and occipital cortical thinning and hypometabolism are parallel to phenoconversion (46, 47). EEG FC matrices showed significantly decreased FC between bilateral ANG and basal ganglia in our study. MRI further consolidated the connection with bilateral CAU, PUT and PAL in MSA and PD, whereas iRBD exhibited significance between ANG.R and contralateral striatum. The examination of DAT uptake illustrated an underlying correlation between EEG disruption and dopaminergic degeneration. Putamen SBR was positively associated with the FC between ANG and striatum in the alpha band which suggested the disrupted alpha FC may be parallel to dopaminergic degeneration. The structural damage and α-synuclein pathophysiology involving ANG have been illustrated in patients with PD and PD dementia (48–50). ANG atrophy and its hypoconnectivity with the occipital cortex are also observed in iRBD with MCI (51, 52). Previous studies suggested gray matter atrophy progressively expanded from basal ganglia to ANG and temporal cortex (53). As a result, our study concluded that ANG played an important role in the process of neurodegeneration.

The groupwise comparisons of iRBD with MSA and PD exhibited significant differences in different frequency bands, which could be attributed to characteristics of different converting directions. The elevation of gamma FC in MSA might be correlated with more severe motor dysfunction since the gamma oscillation is considered to be associated with movement executions. However, the abnormality in the theta band might be associated with motor and cognitive impairment in PD.

There are some limitations which should be acknowledged. First, on account of the cross-sectional design, a direct causal relationship between the EEG/MRI changes and the direction of phenconversion cannot be concluded from this study. Second, the EEG and MRI data of each individual were not recorded simultaneously limited by the instruments. Future studies using simultaneous resting-state MRI and EEG are necessary for mapping the MRI correlates to continuous rhythmic EEG modulations. Third, considering part of the iRBD population completed the DAT scan, the corresponding statistic power had limitations. The DAT images of HC, MSA and PD populations are also important to quantify the relative loss of DAT between different groups. Last, additional validation in larger cohorts is warranted to improve the reliability and reproducibility of the aberrant functional connectomics, and propel the utility of the multimodal biomarkers for clinical diagnosis.

## Conclusion

In summary, our study identified electrophysiologic and neuroimaging profiles in patients with α-synucleinopathies. The alteration of delta spectral power was associated with cognitive performance. EEG FC in the theta and gamma bands were disrupted and correlated with nonmotor symptoms. Decreased alpha FC indicated the severity of motor dysfunction in MSA and PD. Integrating functional EEG and MRI analysis, we found homogenous FC abnormality between ANG and striatum in α-synucleinopathies. The decreased FC was relevant to the DAT deficit in iRBD, suggesting prodromal neurodegeneration. FC between OLF and DLPFC significantly differed in PD and MSA. The multimodal approach integrating spectral and FC features of EEG and MRI may provide reliable methodologies for the discrimination of MSA and PD.

## Data availability statement

The original contributions presented in the study are included in the article/Supplementary material, further inquiries can be directed to the corresponding author/s.

## Ethics statement

The studies involving humans were approved by Ethics Committee of Ruijin Hospital, Shanghai Jiaotong University School of Medicine. The studies were conducted in accordance with the local legislation and institutional requirements. The participants provided their written informed consent to participate in this study.

## Author contributions

CW: Conceptualization, Formal analysis, Investigation, Methodology, Writing – original draft. JH: Data curation, Methodology, Writing – original draft. PL: Methodology, Project administration, Supervision, Writing – review & editing. MZha: Formal Analysis, Methodology, Writing – review & editing. LZ: Methodology, Supervision, Validation, Writing – review & editing. NL: Project administration, Visualization, Writing – review & editing. XZhu: Methodology, Supervision, Writing – review & editing. QY: Project administration, Writing – review & editing. MZho: Methodology, Project administration, Writing – review & editing. XZho: Project administration, Writing – review & editing. HW: Writing – review & editing. YL: Conceptualization, Writing – review & editing. BL: Supervision, Writing – review & editing. JL: Writing – review & editing.

## Supplementary material

Supplementary Figure 1 PSD and FC attributes of EEG analysis in iRBD compared with HC. **(A)** Topological maps representing the global PSD per group within the delta and theta band. **(B)** Comparison of global PSD in different frequency bands between HC and iRBD. **(C)** EEG FC analysis after source reconstruction across different frequency bands and groups. **(D)** wPLI in the theta band showed significant differences. **(E)** Graph theory of Eloc, degree and Eglob in the theta band showed significant differences. **(F)** Relationship between EEG indices and the severity of motor and nonmotor dysfunctions in iRBD.

Supplementary Figure 2 Groupwise comparisons of whole brain EEG FC and corresponding MRI analysis between patients with MSA and iRBD, and between patients with PD and iRBD. **(A)** wPLI in the gamma band showed significant differences between patients with MSA and iRBD. **(B)** wPLI in the theta band showed significant differences between patients with PD and iRBD. **(C)** Circular graph visualizing the brain regions responsible for the EEG-FC discrepancies comparing MSA with iRBD. **(D)** Circular graph visualizing the brain regions responsible for the EEG-FC discrepancies comparing PD with iRBD. **(E)** MRI FC analysis between SOG.R and FFG.R in HC, MSA and iRBD groups. **(F)** MRI FC analysis between OLF.R and SOG.R in HC, PD and iRBD groups.
