# Supplementary material for: Network disruption based on multi-modal EEG-MRI in α-synucleinopathies
Source: Front Neurol. 2024 Aug 22;15:1442851. doi: 10.3389/fneur.2024.1442851 (PMC11374649; doi:10.3389/fneur.2024.1442851)
Supplement: Supplementary file 1 [file Data_Sheet_1.DOCX]

# **Supplementary Materials:**

**Materials and Methods**

**EEG Preprocessing**

A band-pass filter of 0.1–60 Hz and a notch filter of 50 Hz were set using a finite impulse response filter. Channels with excessive noise, drift, or bad connection were interpolated using spherical interpolation. The EEG recording should have less than three electrodes with excessive artifacts, otherwise, the EEG was excluded from the analysis. Detection and rejection of artifacts were completed through Independent Component Analysis using the Infomax algorithm

**MRI acquisition and pre****processing**

The scanning protocol included a high-resolution three-dimensional structural T1-weighted magnetization prepared rapid acquisition gradient-echo (MPRAGE) sequence acquired sagittally (slices=192, field of view=250mm2, thickness=1.0mm, flip angle=9o, voxel size=0.5×0.5×1 mm3, echo time=2.44ms, repetition time=1900ms, and inversion time = 900ms); and a whole-brain echo-planar imaging (EPI) run sensitive to blood oxygen level dependent (BOLD) contrast (slices=36, field of view=192mm2, thickness=3.0mm, flip angle=90o, voxel size=3.0×3.0×3.0 mm3, echo time=22ms, repetition time=2000ms, and inversion time =900ms).

MRI Images were performed using a data processing assistant for resting-state fMRI (DPARSF) 5.4 and Statistical Parametric Mapping (SPM) version 12 (Welcome Department of Cognitive Neurology, London, UK). The first ten time points were discarded. Slice timing and Head motion were also corrected. Images were spatially normalized to the standard MNI 152 space. The image was resampled to 3-mm isotropic voxels and spatially smoothed with a 4 mm full width at half maximum (FWHM) Gaussian kernel. Linear trend and nuisance covariates, including head motions, cerebral fluid, white matter and global average signal were removed. Spurious data were bandpass filtered from 0.01 to 0.08 Hz.

**PET acquisition and preprocessing**

Patients were intravenously injected with ^18^F-FP-CIT at a mean dose of 3.7 MBq/kg body weight. The distribution of dopamine transporter was measured 90 min post-injection. ^18^F-FP-CIT was supplied by Ruijin Hospital, Shanghai Jiao Tong University School of Medicine. Static ^18^F-FP-CIT PET data were acquired in sinogram mode for 15 min with the following parameters: 128 slices per slab, gap 0.5 mm; matrix size 344×344, reconstructed with ordered subsets expectation maximization (OSEM) iterative reconstruction with subsets 21, iterations four and post-filtered with an isotropic full-width at half-maximum Gaussian kernel of 2 mm. Attenuation correction (AC) was performed based on the T1w Dixon images. For patients with iRBD, MRI was performed simultaneously to PET data acquisition.

The PET image was first registered to the corresponding T1w structural image using affine transformation. Subsequently, the T1w image was nonlinearly registered to the standard MNI T1w template. The AAL atlas in the same space of MNI template was warped to the individual PET space by inversely applying the transformation field and matrix above. The registration process was performed using ANTsR.
