# Supplementary figures and images for: Network disruption based on multi-modal EEG-MRI in α-synucleinopathies
Source: Front Neurol. 2024 Aug 22;15:1442851. doi: 10.3389/fneur.2024.1442851 (PMC11374649; doi:10.3389/fneur.2024.1442851)

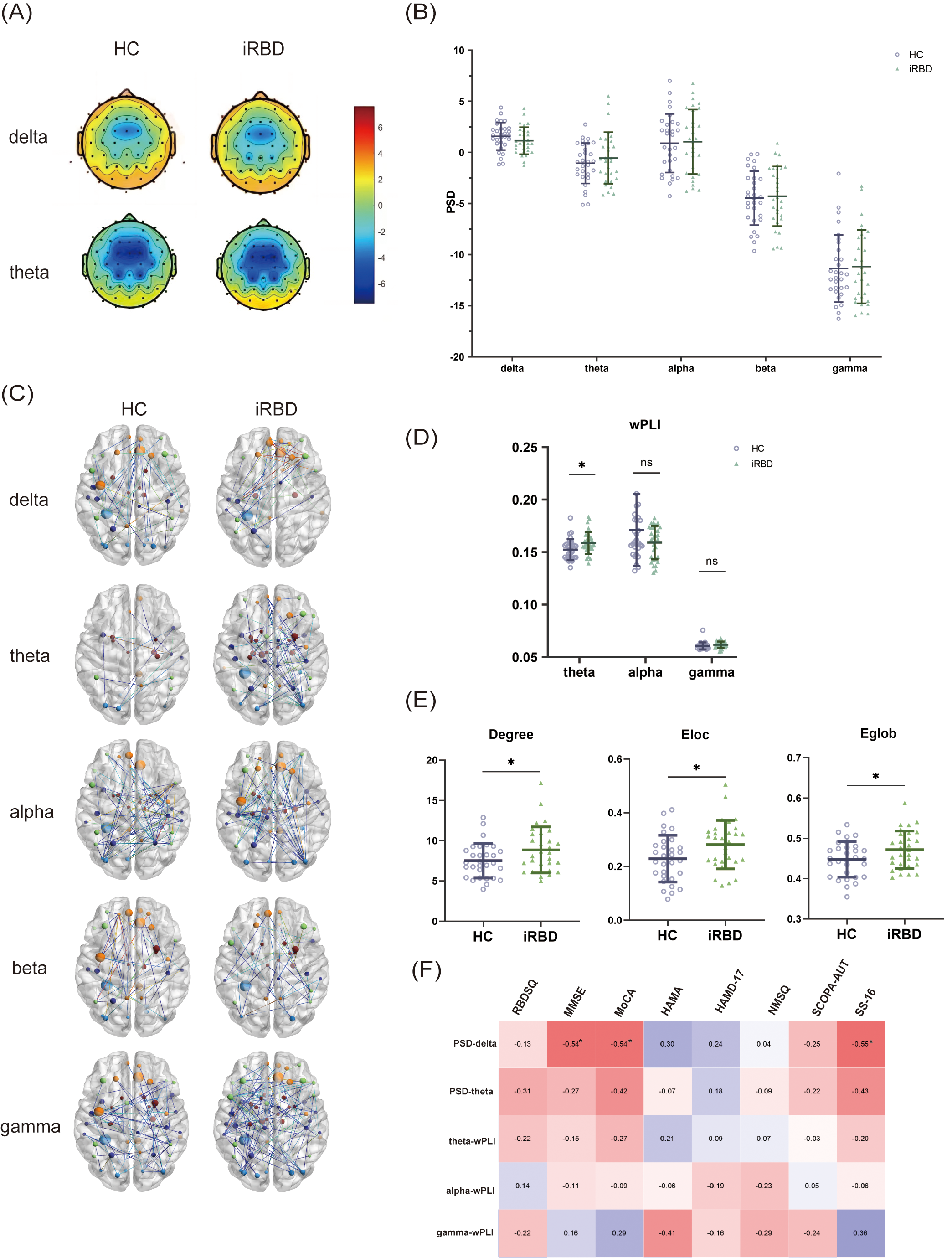

Supplement: Supplementary Figure 1 — PSD and FC attributes of EEG analysis in iRBD compared with HC. (A) Topological maps representing the global PSD per group within the delta and theta band. (B) Comparison of global PSD in different frequency bands between HC and iRBD. (C) EEG FC analysis after source reconstruction across different frequency bands and groups. (D) wPLI in the theta band showed significant differences. (E) Graph theory of Eloc, degree and Eglob in the theta band showed significant differences. (F) Relationship between EEG indices and the severity of motor and nonmotor dysfunctions in iRBD. [file Image_1.TIF]

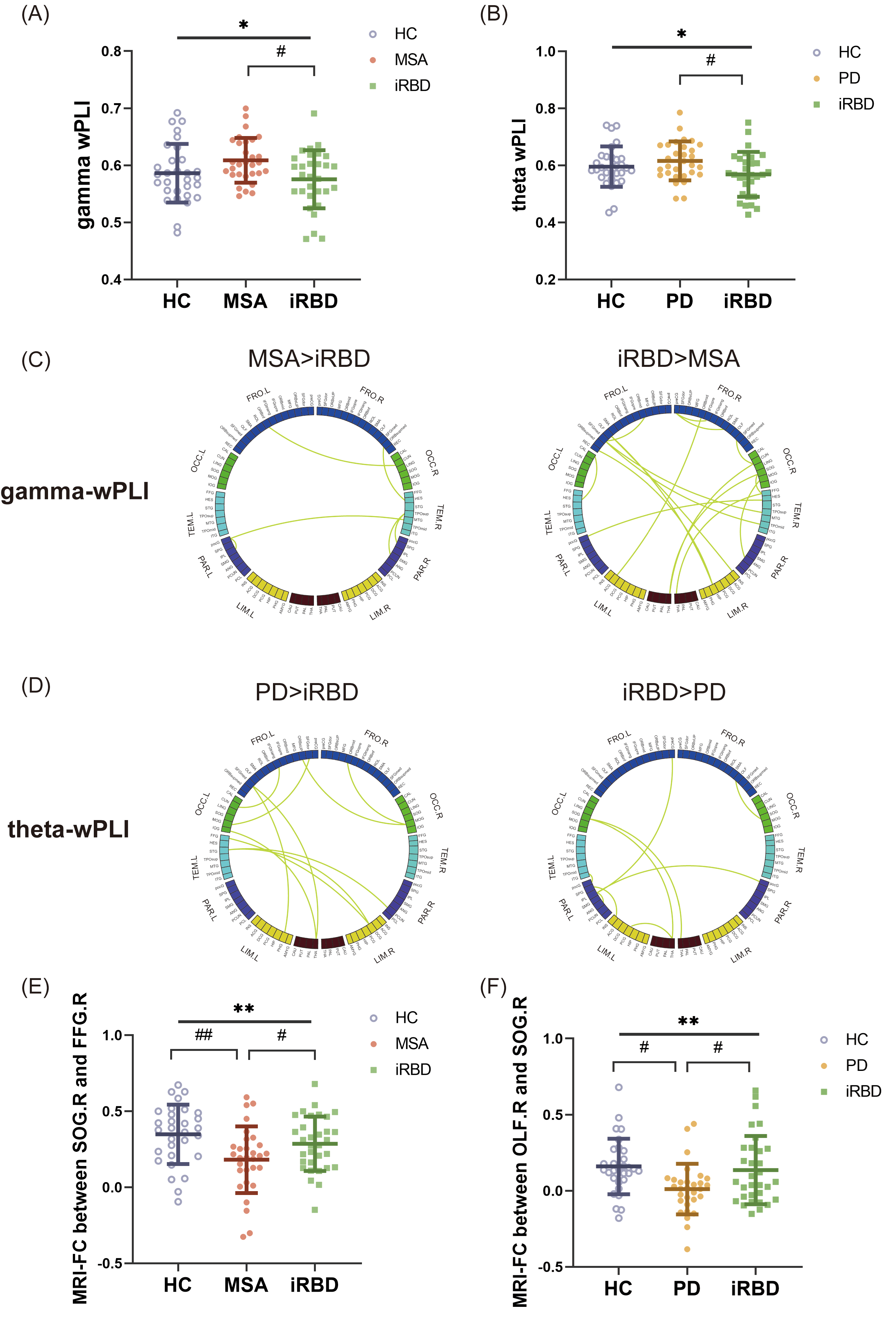

Supplement: Supplementary Figure 2 — Groupwise comparisons of whole brain EEG FC and corresponding MRI analysis between patients with MSA and iRBD, and between patients with PD and iRBD. (A) wPLI in the gamma band showed significant differences between patients with MSA and iRBD. (B) wPLI in the theta band showed significant differences between patients with PD and iRBD. (C) Circular graph visualizing the brain regions responsible for the EEG-FC discrepancies comparing MSA with iRBD. (D) Circular graph visualizing the brain regions responsible for the EEG-FC discrepancies comparing PD with iRBD. (E) MRI FC analysis between SOG.R and FFG.R in HC, MSA and iRBD groups. (F) MRI FC analysis between OLF.R and SOG.R in HC, PD and iRBD groups. [file Image_2.TIF]
